# Catalytic Scalable Synthesis of Polyketides Enabled by Chiral Brønsted Acid‐Catalyzed Asymmetric Allylborations

**DOI:** 10.1002/tcr.202500345

**Published:** 2026-02-15

**Authors:** Shigenobu Umemiya, Masahiro Terada

**Affiliations:** ^1^ Research and Analytical Center for Giant Molecules Graduate School of Science Tohoku University Sendai Miyagi Japan; ^2^ Department of Chemistry, Graduate School of Science Tohoku University Sendai Miyagi Japan

**Keywords:** allylboration, chiral Brønsted acid, organocatalyst, polyketides, scalable synthesis

## Abstract

The scalable synthesis of target molecules is essential for advancing applications, such as evaluating biological activity in pharmaceutical candidates and developing functional materials. In the context of medicinal chemistry, both industrial and academic researchers have achieved the scalable synthesis of diverse natural products and their derivatives, vastly boosting the development of pharmaceuticals. Most established scalable syntheses have conventionally relied on chiral pool approaches or stoichiometric asymmetric methodologies, and the use of catalytic asymmetric strategies has remained limited. In this personal account, we outline recent progress in the development and refinement of asymmetric allylboration reactions catalyzed by chiral Brønsted acids, providing important chiral building blocks that were previously difficult to access. We also describe the catalytic scalable synthesis of potent natural products enabled by the efficient preparation of chiral building blocks utilizing our allylboration reactions.

## Graphical abstract

The scalable synthesis of target molecules is crucial for applications such as biological evaluation in medicinal chemistry and even industrial production. We described recent advances in chiral Brønsted acid–catalyzed asymmetric allylboration, which enables access to previously elusive chiral building blocks, and highlight its application to the scalable catalytic synthesis of biologically active natural products.

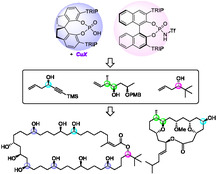

## 1 Introduction

The establishment of efficient synthetic routes to natural products and pharmaceuticals is a crucial challenge in organic synthesis [1, 2, 3, 4, 5, 6]. In particular, the development of concise and scalable synthetic pathways for the sufficient supply of target compounds is a key objective in modern natural product synthesis. This is because a stable and abundant supply of such compounds by chemical synthesis will enable further studies, such as structure–activity relationship (SAR) investigations, detailed mechanistic analyses, clinical research, and even industrial production, thereby broadly impacting both chemical innovation and pharmaceutical research [7].

**FIGURE 1:**
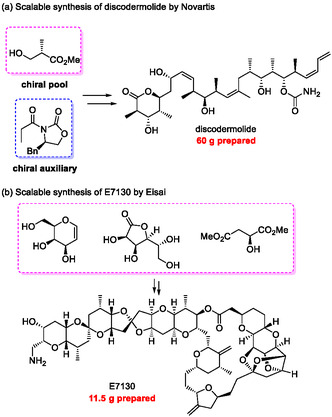
Examples of scalable synthesis of potent natural products by pharmaceutical companies.

Over the past several decades, extensive efforts in organic chemistry have unveiled the remarkable capability of modern organic synthesis to construct highly complex molecular architectures. For instance, in 2004, Novartis accomplished an efficient and scalable total synthesis of discodermolide, a marine natural product exhibiting potent antitumor activity (Figure 1) [8]. They succeeded in preparing 60 g of this compound, which is otherwise scarcely available from natural sources, enabling diverse biological studies. Two decades later, in 2024, Eisai established a scalable synthetic route to E7130, a structurally complex compound related to halichondrin and eribulin. More than 10 g of E7130 was synthesized for use in clinical trials [9].

These achievements highlight the maturity and power of contemporary organic synthesis, which can now provide highly complex biologically active compounds in practical quantities, thereby facilitating the discovery and development of important therapeutics. Not only industries but also academic research groups have achieved the scalable synthesis of diverse natural products [10, 11, 12, 13, 14, 15], and the impact of these accomplishments on the advancement of both organic synthesis and medicinal chemistry is immeasurable.

On the other hand, many of the scalable syntheses reported to date have relied heavily on chiral pool strategies, and the majority of asymmetric reactions employed are stoichiometric reactions utilizing chiral auxiliaries. Although these approaches are well‐established and reliable, they inherently limit access to non‐natural stereochemical motifs and often generate substantial chemical waste because they require more than stoichiometric amounts of chiral sources. Such issues in conventional scalable syntheses frequently escalate manufacturing costs and increase environmental burden, a formidable challenge that must be addressed in the current climate of environmental degradation and global inflation. Furthermore, stoichiometric asymmetric reactions employing chiral auxiliaries often require highly reactive organometallic reagents or those unstable to moisture and oxygen. Consequently, issues remain regarding their handling and safety, particularly in large‐scale experiments conducted at the early stages of synthesis.

Numerous synthetic strategies have been developed to enable the efficient construction of structurally complex natural products. In particular, within the fields of polyketide and macrolide synthesis that are the focus of this account, groundbreaking methodologies have been reported, including Anion Relay Chemistry (ARC) [16], which has been intensively developed by Smith and co‐workers, as well as the iterative organoboron‐based process for the synthesis of 1,3‐polyol motifs recently developed by Aggarwal and co‐workers [17, 18]. While these approaches do not rely on asymmetric catalysts for stereocontrol, they successfully leverage reagent‐controlled strategies to achieve the efficient construction of 1,3‐stereodiols and 1,5‐stereodiols, which are commonly found in polyketides, with exceptionally high stereoselectivity.

As asymmetric catalysis enables the synthesis of large quantities of enantioenriched compounds from only a small amount of a chiral source, it has remained one of the most fundamental topics in organic synthesis for decades [19, 20, 21, 22, 23, 24, 25]. The practical synthesis of enantioenriched natural products greatly benefits from the ability to prepare key intermediates in sufficient quantities through asymmetric catalysis, which lowers overall costs and reduces chemical waste. Furthermore, because the stereochemical outcome of such reactions can be controlled simply by utilizing the catalyst enantiomer, all possible diastereomers can be accessed theoretically. This flexibility makes asymmetric catalysis particularly valuable for SAR studies and biological evaluations in drug research.

**FIGURE 2:**
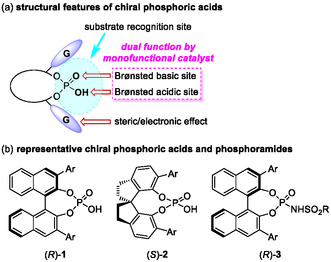
Structures of chiral Brønsted acid catalysts.

In this context, our group has developed a variety of asymmetric transformations catalyzed by chiral Brønsted acids. In particular, chiral phosphoric acids (CPAs) [26, 27, 28, 29, 30, 31, 32, 33, 34, 35, 36] are readily available, exceptionally stable, highly active, and easily recoverable organocatalysts. The structural features of CPA catalysts are summarized in Figure 2a. CPAs exhibit relatively high acidity (pKa 1–2 in H_2_O); the hydroxyl group functions as a hydrogen‐bond donor (acidic site) to activate electrophiles, and the phosphoryl group serves as a hydrogen‐bond acceptor (basic site). In other words, CPAs can be described as a “dual function by a monofunctional catalyst,” because a single functional group operates as both an acid and a base [37]. In addition, the substituents on the side chains can be tuned to modulate steric and electronic effects, thereby creating an effective chiral environment. CPAs achieve a high level of stereochemical control through multiple molecular interactions with substrates, including hydrogen bonding, *π*‐stacking, and other interactions. We have exploited these catalysts to achieve environmentally benign molecular transformations based on chiral Brønsted acid catalysis. Recently, we have focused on developing asymmetric allylboration reactions catalyzed by chiral Brønsted acids, including BINOL‐derived CPA **1**, SPINOL‐derived CPA **2**, and chiral phosphoramide **3** (Figure 2b) [38], with particular emphasis on their applicability to scalable synthesis.

In this personal account, we describe in detail the development of asymmetric allylboration reactions and their application to the efficient and scalable total syntheses of fostriecin, leucascandrolide A macrolactone, and bastimolide A, considered important natural products because of their potent biological activities. Their limited availability from natural sources makes them particularly meaningful targets for catalytic scalable synthesis.

## 2 Chiral Brønsted Acid‐Catalyzed Allylboration Reactions

### 2.1 Background

**SCHEME 1:**
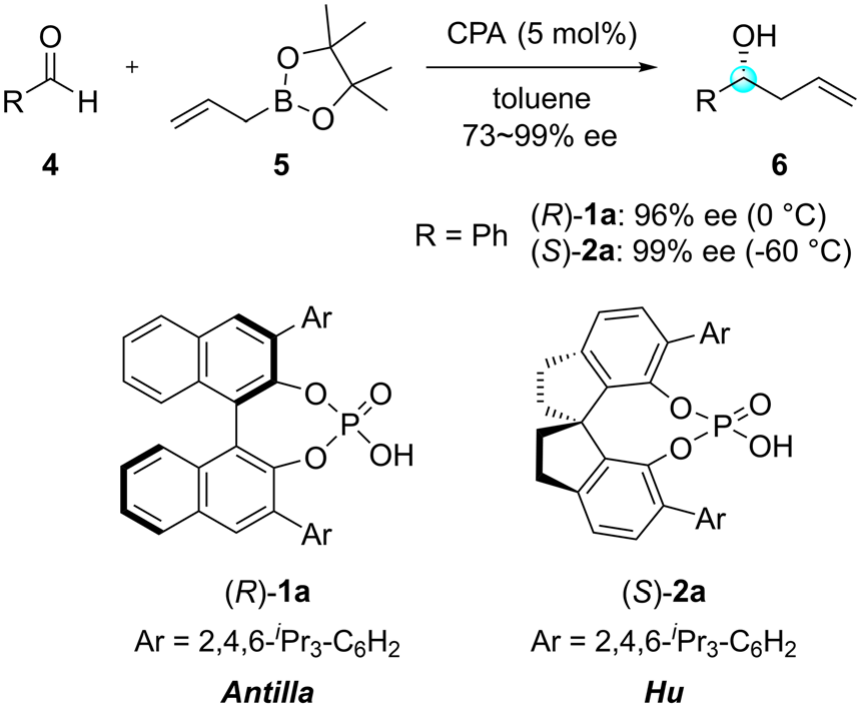
Enantioselective CPA‐catalyzed allylboration reactions by Antilla and Hu.

In 2010, the Antilla group reported the first CPA‐catalyzed enantioselective allylboration reaction (Scheme 1) [39]. They found that the reaction of aldehydes **4** with allylboronic acid pinacol ester (**5**) proceeded smoothly in the presence of BINOL‐derived phosphoric acid **1a** to afford the corresponding homoallylic alcohols in high yields and with excellent enantioselectivities. The reaction proceeded under remarkably mild conditions, providing the desired products with high enantioselectivity when a range of benzaldehyde derivatives and *α*,*β*‐unsaturated aldehydes were employed as substrates. In contrast, a slight decrease in enantioselectivity was observed when aliphatic aldehydes, particularly those bearing a cyclohexyl substituent, were used. In 2012, Hu and coworkers reported an enantioselective allylation reaction mediated by SPINOL‐derived catalyst **2a** (Scheme 1) [40]. This catalyst exhibited excellent enantioselectivity even with substrates that were previously problematic under BINOL‐derived CPA **1a**, thereby expanding the accessible substrate scope of CPA‐catalyzed allylboration reactions.

**FIGURE 3:**
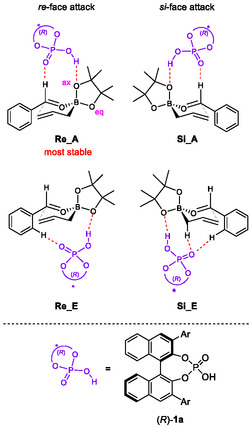
Transition state models of CPA‐catalyzed allylborations.

In 2012, Goodman and coworkers reported a computational study on the transition state analysis of the asymmetric allylboration reaction developed by the Antilla group using DFT calculations (Figure 3) [41]. In this work, it was proposed that the acidic hydroxyl group of CPA forms a hydrogen bond with the axial oxygen of the boronate, while the phosphoryl oxygen interacts with the formyl hydrogen of benzaldehyde. In this account, the transition state involving *re*‐face attack with axial coordination is denoted as **Re_A**, whereas the corresponding transition state for *si*‐face attack is referred to as **Si_A**. Furthermore, Houk and coworkers also reported a computational investigation into transition states featuring equatorial coordination of the CPA‐catalyzed allylboration in 2013 [42]. They reported that the transition state involving *si*‐face attack with equatorial coordination (**Si_E**) is more stable than the axial one (**Si_A**). Prompted by these seminal reports, research on asymmetric allylation reactions catalyzed by CPA has been extensively pursued from both experimental and theoretical perspectives.

Over the past decade, several natural products have been synthesized using CPA‐catalyzed asymmetric allylation as a key transformation [43, 44, 45, 46, 47, 48, 49, 50, 51, 52, 53]. The target molecules are relatively simple, often containing benzaldehyde or *α*,*β*‐unsaturated aldehyde motifs. Conventional catalysts have shown limited substrate scope, and the range of natural products accessible through such methods has been restricted to relatively simple structures. The reported CPA‐catalyzed asymmetric allylation was insufficiently broad for the synthesis of various natural products, posing a primary challenge at the outset of our study [54, 55]. We envisioned that the appropriate utilization of CPA's features, including high catalyst activity and ease of recovery, would be essential to achieve ideal scalable syntheses. CPA‐mediated organocatalytic processes for preparing essential building blocks are ideally suited for the scalable synthesis of complex natural products. Thus, we initiated studies to refine CPA‐catalyzed enantioselective allylboration reactions and used them as key steps in the total synthesis of natural products.

**SCHEME 2:**
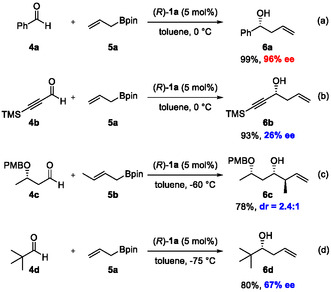
Conventional asymmetric allylboration catalyzed by CPA and unsuccessful examples.

During the course of investigations, it became evident that certain substrates were not compatible with conventional reaction conditions (Scheme 2). For example, aldehydes bearing a triple bond **4b** (Scheme 2b), which serve as a useful functional group for further transformations, showed markedly diminished enantioselectivity (26% ee) when subjected to established BINOL‐derived CPA (*R*)‐**1a**. The diastereoselective allylboration of enantioenriched *β*‐alkoxy aldehyde **4c** afforded a product with poor selectivity (Scheme 2c). Moreover, sterically hindered aldehyde, such as pivalaldehyde **4d**, was also not a suitable substrate for the conventional CPA‐catalyzed allylboration reaction (Scheme 2d). These aldehydes are synthetically versatile compounds commonly employed in natural product synthesis and serve as valuable chiral building blocks. Considering that CPA‐catalyzed allylation proceeds under exceptionally mild conditions, it may be highly advantageous for the synthesis of functionally dense and complex molecules, particularly polyketide natural products. Motivated by this prospect, we set out to develop efficient and scalable synthetic routes to the representative compounds shown in Scheme 2, as a foundation for achieving truly catalytic and scalable syntheses of related natural products.

### 2.2 Catalytic Enantioselective Allylboration of Acetylenic Aldehydes by Chiral Phosphoric Acid/Transition Metal Cooperative Catalysis

**FIGURE 4:**
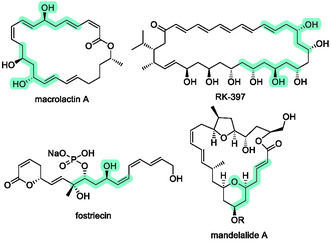
Examples of natural products in which enantioenriched propargyl alcohols serve as key intermediates.

Enantioenriched propargyl alcohols are versatile synthetic building blocks for the preparation of natural products and pharmaceuticals, as the alkyne moiety can be readily transformed into diverse functional groups [56, 57, 58]. Among them, propargyl alcohol **6b**, obtained through the asymmetric allylation of silyl‐substituted acetylenic aldehydes, is a particularly valuable intermediate for the construction of structurally complex molecules [59, 60, 61, 62]. Indeed, numerous total syntheses of natural products have employed enantioenriched alcohol **6b** as a pivotal intermediate (Figure 4, light green highlight). The development of efficient catalytic methods to access enantioenriched homoallylic propargyl alcohol **6b** remains a significant objective in synthetic organic chemistry, as it is expected to streamline the synthesis of architecturally intricate targets.

The catalytic enantioselective allylation of acetylenic aldehydes has attracted significant attention owing to its utility in natural product synthesis. For instance, catalytic enantioselective allylation of acetylenic aldehydes has attracted significant attention due to its utility in natural product synthesis. For instance, Hall and coworkers reported an enantioselective allylation of trimethylsilyl (TMS)‐substituted acetylenic aldehydes **4b** catalyzed by a chiral SnCl_2_/F‐Vivol‐8 complex [63]. Carter's group applied the Keck allylation protocol in the total synthesis of mandelalide A to obtain enantioenriched propargyl alcohols **4b** with moderate enantioselectivity [64]. Recently, Krische and coworkers later developed an Ir‐catalyzed reductive coupling of acetylenic aldehydes with allyl acetates, efficiently furnishing TIPS‐substituted propargyl alcohols in excellent yield and enantioselectivity [65]. However, TMS‐substituted acetylenic aldehydes decomposed under the Krische's conditions, even though the TMS group at the alkyne terminus is synthetically advantageous because it can be selectively removed under mild conditions in the presence of other silyl protection groups.

To identify the optimal conditions for the enantioselective allylation of an acetylenic aldehyde, TIPS‐substituted aldehyde **4e** was chosen as the model substrate (Table 1). The reaction of **4e** with allylboronic acid pinacol ester **5a** was performed at 0°C in the presence of the BINOL‐derived CPA (*R*)‐**1a** (Ar = 2,4,6‐^i^Pr_3_‐C_6_H_2_). Aldehyde **4e** was completely consumed within 1 min, affording the desired product in excellent yield but with low enantioselectivity (entry 1). SPINOL‐derived CPA (*R*)‐**2a** (Ar = 2,4,6‐^i^Pr_3_‐C_6_H_2_) was highly effective for the enantioselective allylation of aldehydes bearing phenyl‐ or alkyl‐substituted acetylenic groups^10^: it promoted the allylation of the TIPS‐substituted aldehyde efficiently, providing (*S*)‐**6e** in excellent yield but with moderate enantioselectivity (entry 2). Next, a range of transition metal salts that could potentially coordinate to the alkyne moiety of **4e** were screened (entries 3–10). The addition of CuBr enhanced the enantioselectivity from 73% ee to 86% ee. A comparable cooperative effect was observed with CuI, furnishing the desired product with good enantioselectivity (entry 4). In contrast, the use of CuTC or [CuOTf]_2_·PhMe failed to provide any improvement, giving (*S*)‐**6e** with only 36% ee or 74% ee, respectively (entries 5 and 6). Further experiments employing Ag(I), Bi(III), Rh(II), and Pt(II) additives revealed that these metals had no beneficial effect in the present enantioselective allylation (entries 7–10). Finally, conducting the reaction at –60°C under the cooperative CPA/CuBr catalytic system using (*R*)‐**2a** delivered the product in 98% yield with perfect enantioselectivity (>99% ee) (entry 11). In the absence of CuBr, somewhat lower selectivity (96% ee) was obtained relative to the cooperative system (entry 11 vs 12). These observations clearly demonstrated that the combination of (*R*)‐**2a** and CuBr enhances the enantioselectivity of this allylation even at low temperature.

**TABLE 1:** Optimization of allylboration reaction with acetylenic aldehyde **4e**.^a^

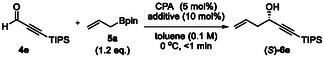				
Entry	CPA	Additive	Yield/%^b^	Ee/%^c^
1	(*R*)‐**1a**	none	93	−26^d^
2	(*R*)‐**2a**	none	95	73^e^
3	(*R*)‐**2a**	CuBr	99	86
4	(*R*)‐**2a**	CuI	98	80
5	(*R*)‐**2a**	CuTC	93	36
6	(*R*)‐**2a**	[CuOTf]_2_·PhMe	95	74
7	(*R*)‐**2a**	AgOBz	90	−4
8	(*R*)‐**2a**	Bi(OAc)_3_	96	58
9	(*R*)‐**2a**	Rh_2_(OAc)_4_	94	66
10	(*R*)‐**2a**	PtCl_2_(PPh_3_)_2_	96	64
11^f^	(*R*)‐**2a**	CuBr	98	>99
12^f^	(*R*)‐**2a**	none	97	96
13^f^^,^ g	(*R*)‐**2a**	CuBr	92	>99

a Unless otherwise noted, all reactions were carried out using 0.20 mmol of **4e**, 0.24 mmol of **5a**, and 0.010 mmol of CPA catalyst (5 mol%) in toluene.

b Isolated yield.

c Enantiomeric excess was determined by chiral stationary phase HPLC analysis.

d (*R*)‐**6e** was obtained.

e (*S*)‐**6e** was obtained.

f The reaction was performed at −60°C for 24 h.

g Aldehyde **4b** was employed.

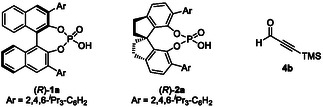

With the optimized conditions established, TMS‐substituted acetylenic aldehyde **4b** was reacted with allylboronic acid **5a** to afford the desired product in 92% yield and >99% ee without any observable side reactions, such as substrate decomposition (entry 13).

**SCHEME 3:**
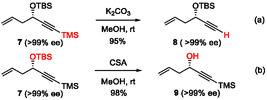
Selective deprotection of silyl groups.

Moreover, the synthetic versatility of the allylation product obtained from TMS‐substituted acetylenic aldehyde **4b** was demonstrated through selective desilylation under basic or acidic conditions (Scheme 3). The TMS group attached to the alkyne moiety was selectively cleaved under mild basic conditions (K_2_CO_3_/MeOH), whereas the secondary *t*‐butyldimethylsilyl (TBS) ether was intact (Scheme 3a). Conversely, treatment with 10‐camphorsulfonic acid (CSA) in methanol led to deprotection of the secondary TBS ether, giving the corresponding alcohol without affecting the terminal TMS substituent (Scheme 3b). Throughout both transformations, the enantiomeric purity of the product remained unchanged. These complementary deprotection protocols underscore the importance of TMS‐substituted propargyl alcohol as an intermediate for late‐stage functionalization in the synthesis of complex molecules.

**SCHEME 4:**
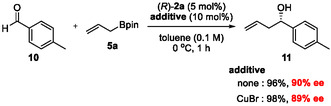
Effect of CuBr in the CPA‐catalyzed allylboration of 4‐methylbenzaldehyde **10**.

Next, to determine whether the effect of copper was specific to substrates bearing a triple bond, we conducted a control experiment, as shown in Scheme 4. When 4‐methylbenzaldehyde (**10**) was reacted with **5a** in the presence of catalyst (*R*)‐**2a** and CuBr, desired product **11** was obtained, with enantioselectivity almost identical to that observed in the absence of CuBr. This suggests that the copper reagent does not interact with catalyst **2a** or boron reagent **5a**. Instead, it coordinates specifically to the alkyne moiety of acetylenic aldehydes. Palomo's group reported an asymmetric aldol reaction of acetylenic aldehydes catalyzed by an organocatalyst–transition metal cocatalyst system, achieving high levels of stereoinduction [66]. They attributed the enhanced diastereoselectivity to metal–alkyne coordination, which increases steric congestion near the reacting center. Similarly, in our system, CuBr was presumed to coordinate to the alkyne moiety of the aldehyde, enforcing an equatorial orientation of the alkyne substituent in the six‐membered transition state (TS). This coordination should simultaneously destabilize TS of the minor reaction pathway in which the alkyne group occupies an axial position, leading to enhanced enantioselectivity.

### 2.3 Catalytic Asymmetric Allylboration of *β*‐Alkoxy Aldehyde by CPA/CuCl Cocatalyst System for the Construction of 1,3‐*Syn* Diol Structures

Because enantioenriched 1,3‐*syn* diol motifs are commonly found in natural products and pharmaceuticals, such as Lipitor, numerous research groups have developed efficient synthetic methods for their construction [67, 68, 69]. Diastereoselective aldol reactions [70] and hemiacetalization/intramolecular oxy‐Michael reactions, which enable the catalytic formation of the 1,3‐*syn* diol framework [71, 72, 73, 74, 75, 76], have been reported by several research groups. Whereas chelation‐controlled 1,3‐*anti*‐selective allylation reactions [77, 78] are well established, catalytic and asymmetric allylation reactions that provide 1,3‐*syn*‐diol products with high diastereoselectivity remain rare and underexplored. In this context, we envisioned that CPA catalysis would promote 1,3‐*syn*‐selective asymmetric allylation, a challenging task with conventional methods.

We investigated the diastereoselective crotylation of aldehyde **12** catalyzed by SPINOL‐derived CPA (*R*)‐**2a** (Ar = 2,4,6‐^i^Pr_3_‐C_6_H_2_), as shown in Table 2. As a preliminary experiment, the reaction was carried out in the absence of any additive at –60°C, affording the crotylation product in 80% yield as a mixture of diastereomers (dr = 2.8:1, entry 1). Unfortunately, the diastereoselectivity was moderate, likely due to the influence of the stereogenic center located at the *β*‐position of the aldehyde, although the desired 1,3‐*syn* isomer was obtained predominantly. Subsequently, several metal additives capable of coordinating to the alkoxy substituent of aldehyde **12** were examined. The use of LiCl had no beneficial impact, providing a result similar to that in entry 1. Notably, the introduction of ZnBr_2_ or CuBr as an additive led to a modest enhancement in diastereoselectivity. To our delight, CuCl afforded significantly improved selectivity (dr = 7.8:1, entry 5). Both yield and diastereomeric ratio were further increased when the concentration of toluene solution was increased from 0.1 to 0.5 M, delivering the product in 90% yield with dr = 10.0:1 (entry 6). In contrast, replacing the *p*‐methoxybenzy (PMB) group with a TBS group, which cannot coordinate to metal additives, resulted in a pronounced decrease in diastereoselectivity (dr = 1.5:1, entry 8). This observation indicates that the coordination between CuCl and the PMB moiety is crucial in achieving high diastereoselectivity. Finally, the optimized reaction conditions were applied on a gram scale to produce the desired product in 95% yield without erosion of diastereoselectivity (entry 9).

**TABLE 2:** Optimization of reaction conditions for diastereoselective crotylation^a^

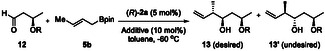				
Entry	R	Additive	Yield /%^b^	**13**:**13’**
1	PMB	none	80	2.8:1
2	PMB	LiCl	79	2.5:1
3	PMB	ZnBr_2_	68	3.6:1
4	PMB	CuBr	83	4.0:1
5^c^	PMB	CuCl	72	7.8:1
6^d^	PMB	CuCl	90	10.0:1
7	TBS	none	65	1.6:1
8	TBS	CuCl	68	1.5:1
9^e^	PMB	CuCl	95	11.8:1

a Unless otherwise noted, all reactions were carried out using 0.2 mmol of **12**, 0.24 mmol of **5b**, and 0.01 mmol of catalyst (5 mol%) in toluene (0.1 M).

b Isolated yield.

c Diastereomeric ratio was determined by ^1^H NMR measurement.

d The reaction was performed in 0.5 M.

e The reaction was performed on a 2.0 g scale using the same conditions as those of entry 6.

### 2.4 Chiral Brønsted Acid‐Catalyzed Enantioselective Allylboration of Sterically Hindered Aldehydes Enabled by Multiple Hydrogen Bonding Interactions

**FIGURE 5:**
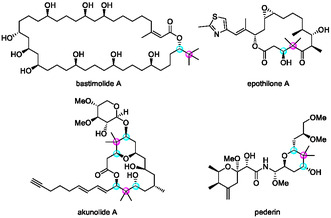
Examples of natural products possessing an enantioenriched secondary alcohol with a neighboring quaternary carbon.

Enantioenriched secondary alcohols bearing a sterically demanding substituent, particularly containing a quaternary carbon adjacent to a stereogenic center, are key structural motifs in numerous biologically active natural products. Many macrolides, for instance, possess this type of chiral secondary alcohol (Figure 5) [79, 80, 81, 82, 83]. Owing to their potent biological activities and architecturally intricate frameworks, these natural products have inspired extensive synthetic efforts, leading to major advances in asymmetric methodology over the past decades.

Various strategies have been developed for constructing a stereogenic center of a secondary alcohol adjacent to a quaternary carbon, ranging from stoichiometric reactions to catalytic asymmetric methods. Whereas stoichiometric and enzymatic approaches are highly reliable, the demand for more efficient and sustainable catalytic processes has driven the development of enantioselective C—C bond‐forming reactions that achieve high selectivity under mild, environmentally benign conditions, emphasized in green and sustainable chemistry [84, 85, 86]. We therefore investigated whether this sterically demanding enantioenriched alcohol could be constructed through a CPA‐catalyzed asymmetric allylation reaction.

In the allylation reaction of pivalaldehyde (**4d**) with allyl boronic reagent **5a**, BINOL‐based CPA (*R*)‐**1a**, a conventional CPA catalyst for asymmetric allylboration reactions, provided product **6d** in good yield with moderate enantioselectivity (Table 3, entry 1). When more acidic catalyst (*R*)‐**1b** possessing a C_6_F_5_ group was employed, both reaction rate and selectivity decreased significantly, suggesting that excessive acidity does not affect catalytic performance directly (entry 2). Next, we employed SPINOL‐derived catalyst (*S*)‐**2a** (TRI*P* = 2,4,6‐^i^Pr_3_‐C_6_H_2_), which was reported to exhibit the best performance in conventional enantioselective allylboration [40]. The reaction between aldehyde **4d** and **5a** catalyzed by (*S*)‐**2a** at −75°C furnished the corresponding product in good yield but with poor enantioselectivity (40% ee, entry 3). These observations indicate that conventional CPAs are ineffective for sterically demanding aldehydes. From our previous studies on the development of a chiral phosphoramide‐catalyzed intramolecular S_*N*_2′ reaction, a phosphoramide catalyst bearing a C_6_F_5_ substituent on the sulfonamide group was found to deliver promising results [87]. Accordingly, we next evaluated catalyst (*R*)‐**3a** featuring a C_6_F_5_SO_2_NH moiety for the allylation of **4d** with **5a** (entry 4). To our delight, the reaction proceeded efficiently, affording the desired product in 98% yield with good enantioselectivity (84% ee). Screening other substituents on the catalyst nitrogen atom revealed that (*R*)‐**3b** containing a triflyl (SO_2_CF_3_, Tf) group exhibited superior reactivity (entry 5), providing a product in quantitative yield with higher enantioselectivity (95% ee) than that obtained with (*R*)‐**3a**. No product was formed when catalyst (*R*)‐**3c** possessing a SO_2_Me substituent was used (entry 6). This result clearly indicates that the nature of the sulfonamide group plays a crucial role in both reactivity and selectivity. No reaction occurred when more acidic thiophosphoramide catalyst (*R*)‐**14** was employed instead of phosphoramide (*R*)‐**3b** (entry 7). The results suggest that the acidity of the catalyst does not directly correlate with either its catalytic activity or the stereochemical outcome in the chiral Brønsted acid‐catalyzed allylation of aldehydes using allylboronic reagents. SPINOL‐derived phosphoramide (*S*)‐**15** also exhibited no activity, likely because of steric restriction around the chiral cavity (entry 8). After a thorough investigation of reaction parameters, including solvent, temperature, and concentration, the conditions employing catalyst (*R*)‐**3b** (entry 5) were identified as optimal. Furthermore, when catalyst loading was reduced to 0.5 mol% in a gram‐scale experiment, the allylation proceeded smoothly to completion, furnishing the desired product quantitatively without any decrease in enantioselectivity (entry 9).

**TABLE 3:** Optimization of reaction conditions for the allylboration of pivalaldehyde.^a^

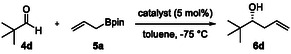				
Entry	Catalyst	Time/h	Yield/%^b^	Ee/%
1	(*R*)‐**1a**	72	80	67
2	(*R*)‐**1b**	72	38	39
3	(*R*)‐**2a**	72	90	40
4	(*R*)‐**3a**	72	98	84
5	(*R*)‐**3b**	24	quant.	95
6	(*R*)‐**3c**	72	no reaction	nd
7	(*R*)‐**14**	72	no reaction	nd
8	(*S*)‐**15**	72	no reaction	nd
9^c^	(*R*)‐**3b**	48	95	95

a Unless otherwise specified, all reactions were carried out using 0.20 mmol of **4d**, 0.24 mmol (1.2 eq.) of **5a**, and 0.010 mmol (5 mol%) of catalyst.

b Isolated yield.

c Catalyst loading was 0.5 mol%, and 2 g of pivalaldehyde was employed. TRI*P* = 2,4,6‐^*i*^Pr_3_‐C_6_H_2_, nd = not determined.

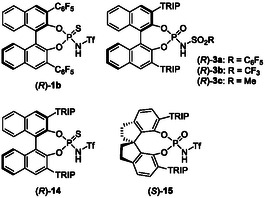

**FIGURE 6:**
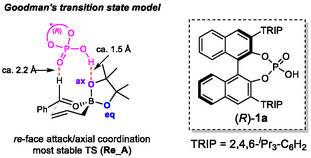
Transition state model of CPA‐catalyzed allylboration by Goodman.

Our profound interest in the origin of the exceptional effectiveness of the phosphoramide catalyst in the present reaction prompted us to perform TS analyses using DFT calculations. Goodman and Houk independently conducted comprehensive computational investigations to uncover the reaction mechanism underlying the CPA‐catalyzed allylboration of benzaldehyde [41, 42]. The lowest energy TS in the allylboration reaction is illustrated in Figure 6. In this model, catalyst (*R*)‐**1a** does not activate the carbonyl group of benzaldehyde through direct protonation. Instead, it stabilizes a six‐membered TS through two distinct hydrogen‐bonding interactions. One occurs between the acidic hydroxyl proton and the axial oxygen atom of the boronic ester (P—O—H···O—B), and the other involves the phosphoryl oxygen and the formyl hydrogen atom of benzaldehyde (*P*=O···H—C). Indeed, DFT calculations indicate that the P—O—H···O—B distance is approximately 1.5 Å, confirming that this interaction involves hydrogen bonding rather than protonation. These computational findings rationalize the reduced reactivity of highly acidic CPA (*R*)‐**1b** (Table 3, entry 2), showing that increased acidity does not necessarily contribute to stabilizing TS of the major reaction pathway.

To gain further mechanistic insight into our reaction system, we conducted DFT studies on the enantioselective allylation of pivalaldehyde (**4d**) catalyzed by chiral phosphoramide (*R*)‐**3b**, which afforded (*R*)‐**6d**. The experimental results showed that the *re*‐face attack pathway is favored over the *si*‐face attack.

**FIGURE 7:**
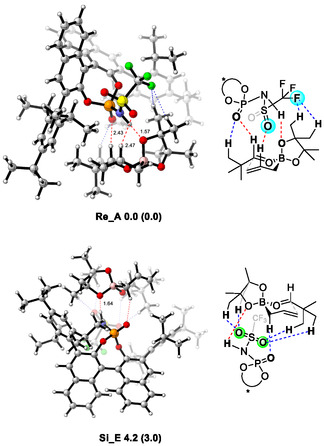
Optimized transition states of **Re_A** and **Se_E** at the M06‐2X/6‐31G(d) level of theory. Bond lengths are given in Å. Values enclosed in parentheses are energies relative to “**Re_A**” calculated by MN15/6‐31g(d)+SDM (toluene). Energy differences are given in kcal/mol.

On the basis of previous studies by Goodman and Houk, four possible TSs were calculated, corresponding to *re*‐ and *si*‐face attacks through both axial and equatorial coordinations. Geometry optimizations were performed using Gaussian 16 at the M06‐2X/6‐31G(d) level of theory. The most stable TS (major TS) was determined to be **Re_A**, which means *re*‐face attack in the axial coordination mode (Figure 7). On the other hand, *si*‐face attack in the equatorial coordination mode, namely, **Si_E** (minor TS), was associated with the formation of the minor enantiomer. Thus, **Re_A** and **Si_E** represent the major and minor reaction pathways, respectively. Similar to conventional CPA‐catalyzed allylborations, the most stable TS, namely the major TS (**Re_A**), features two key hydrogen‐bonding interactions, namely, the phosphoryl oxygen of the catalyst interacts with the aldehydic proton (2.43 Å), and the N—H group of the catalyst forms a hydrogen bond with axial boronate oxygen (1.57 Å). Moreover, the energy difference between the major TS (**Re_A**) and the minor TS (**Si_E**) was calculated to be 4.2 kcal/mol, which was refined to 3.0 kcal/mol after single‐point calculations at the MN15/6‐31G(d)+SMD(toluene) level [88]. These energy difference values are consistent with the experimentally determined enantiomeric excess of 95%, corresponding to a theoretical difference of ca. 2.2 kcal/mol. In **Re_A**, the N—H···O—B hydrogen bond length (1.57 Å) remains essentially unchanged from that calculated in the CPA‐catalyzed allylboration systems, suggesting that the higher acidity of the phosphoramide catalyst does not significantly strengthen this hydrogen bond. Interestingly, in addition to the two principal hydrogen bonds, **Re_A** exhibits additional weak attractive interactions, in which the sulfonyl oxygen interacts with the olefinic C—H bond (S = O···H—C, 2.47 Å), and the CF_3_ group forms two C—F···H—C interactions (2.25 and 2.57 Å) with the pinacol methyl hydrogens. In contrast, **Si_E** lacks both *P* = O···H—C and C—F···H—C interactions. Similarly, these fluorine–hydrogen interactions are absent in other minor TSs (**Re_E** and **Si_A**). Therefore, the calculations indicate that the low stability of the minor TSs arises primarily from the absence of multiple attractive interactions rather than from steric congestion. These results suggest that the sum of several hydrogen‐bonding interactions, including the relatively weak S = O···H—C and C—F···H—C interactions that are nonexistent in conventional CPA‐catalyzed allylborations, plays a critical role in stabilizing the major TS, which accounts for the excellent enantioselectivity observed in this system [89].

## 3 Synthesis of Biologically Active Natural Products by Using Chiral Brønsted Acid‐Catalyzed Allylborations

**FIGURE 8:**
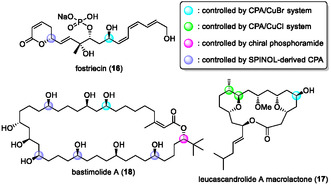
Structures of completed natural products based on chiral Brønsted acid‐catalyzed allylborations.

We next demonstrated the efficient syntheses of three biologically active natural products utilizing three distinct asymmetric allylation reactions developed in our laboratory. Their structures are shown in Figure 8 [90, 91, 92]. The stereocenters constructed by asymmetric allylborations are color‐coded in blue, green, and purple, respectively. For macrolides leucascandrolide A macrolactone (**17**) and bastimolide A (**18**), we aimed for scalable syntheses by employing catalytic reactions wherever possible, thereby establishing routes that facilitate material accessibility. Consequently, we achieved gram‐scale preparation of key intermediates for both syntheses. The details of the synthesis for each of the three compounds are described below.

### 3.1 Concise Formal Synthesis of Fostriecin

Fostriecin (**16**) is a secondary metabolite produced by *Streptomyces pulveraceus* isolated from a Brazilian soil sample [93, 94]. Fostriecin and structurally related natural products constitute an important class of compounds that exhibit potent biological activities against leukemia as well as lung, breast, and ovarian cancer cells [95]. This natural product acts as a selective inhibitor of serine/threonine protein phosphatases, showing remarkable potency toward PP2A (IC_50_ = 1.5 nM) and PP4 (IC_50_ = 3 nM). However, its clinical development has been hampered by chemical instability and the difficulty of obtaining material with consistent purity from natural sources. Thomasi and coworkers subsequently isolated two new phosphorylated natural products, phosdiecins A and B [96]. Krische and coworkers accomplished the first total synthesis of phosdiecin A through an enantioselective carbonyl reductive coupling, confirming the originally proposed structure and enabling evaluation of its biological activity [97]. These findings underscore the value of developing efficient synthetic strategies for fostriecin and its congeners, as such strategies would allow access to these compounds and their analogs in high purity and sufficient quantity for further biological studies.

**SCHEME 5:**
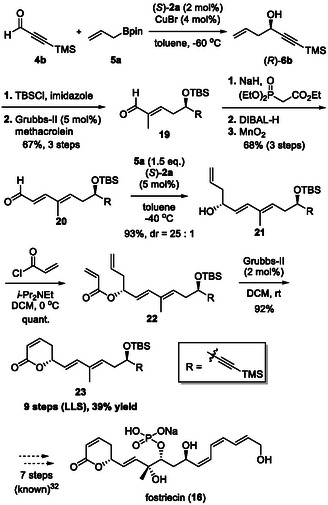
Formal synthesis of fostriecin (**16**).

Our synthesis of fostriecin (**16**) commenced with enantioselective allylboration using the CPA/CuBr cocatalyst system. The enantioselective allylation of TMS‐substituted acetylenic aldehyde **4b** with allylboronic ester **5a** was carried out under the optimized conditions, affording desired homoallylic alcohol (*R*)‐**6b** with excellent enantioselectivity (Scheme 5). Notably, the reaction proceeded smoothly with only 2 mol% of catalyst (*S*)‐**2a** and 4 mol% of CuBr. Subsequent TBS protection, followed by olefin cross‐metathesis using methacrolein in the presence of 5 mol% Grubbs‐II catalyst, afforded corresponding *α*,*β*‐unsaturated aldehyde **19** in good yield. The obtained aldehyde was then subjected to three transformations—the Horner–Wadsworth–Emmons reaction, reduction with DIBAL‐H, and oxidation—giving *α*,*β*‐*γ*,δ‐unsaturated aldehyde **20** in 68% yield in three steps. Treatment of aldehyde **20** with **5a** in the presence of (*S*)‐**2a** provided allylation product **21** in 93% yield with excellent diastereoselectivity. Alcohol **21** was treated with acryloyl chloride under basic conditions, and subsequent ring‐closing metathesis using Grubbs II catalyst afforded *α*,*β*‐unsaturated lactone **23**. This McDonald's intermediate **23** was thus obtained in 39% overall yield in nine steps from commercially available aldehyde **4b** through CPA‐catalyzed enantioselective allylboration [98]. This synthetic route eliminated nine steps from the overall sequence and significantly enhanced the total yield compared with McDonald's synthesis, which required 18 steps and provided intermediate **23** in 18.4% overall yield. During that period, 15 total and formal syntheses of fostriecin have been reported, comprising sequences ranging from 19 to 34 steps (longest linear sequence, LLS). The present synthetic strategy can theoretically deliver the target molecule in only 16 steps. Very recently, the Krische group reported an elegant formal synthesis of fostriecin in 16 steps by utilizing several transition metal‐catalyzed transformations as key steps [99].

### 3.2 Scalable Total Synthesis of Leucascandrolide A Macrolactone

**SCHEME 6:**
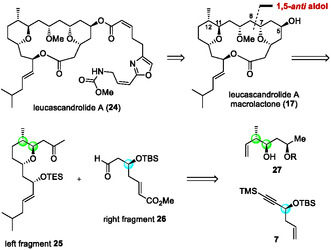
Retrosynthetic analysis of leucascandrolide A macrolactone (**17**).

Leucascandrolide A (**24**) is a highly oxygenated 18‐membered macrolactone bearing eight stereogenic centers, two trisubstituted tetrahydropyran rings, and an oxazole‐containing side chain at C5 (Scheme 6) [100]. This complex macrolide exhibits potent cytotoxicity toward human KB (IC_50_ = 0.05 μM) and P388 leukemia cells (IC_50_ = 0.25 μM), as well as antifungal activity against *Candida albicans*. Despite its remarkable bioactivity, the limited natural availability of leucascandrolide A has hampered detailed SAR studies. Several research groups have tackled its total synthesis. Leighton and coworkers achieved the first total synthesis of leucascandrolide A [101]. Catalytic asymmetric reactions have recently emerged as attractive alternatives to stoichiometric methods in the context of sustainable synthesis. The total syntheses of **24** and its macrolactone **17**, a common intermediate of **24**, employing catalytic asymmetric transformations, were later accomplished by Carreira, Wipf, Paterson, Panek, Yadav, and Hong, who independently demonstrated the power of enantioselective catalysis for the construction of complex molecules [102, 103, 104, 105, 106, 107]. Although these routes achieved respectable overall yields (0.22% – 8.12%), the inclusion of catalytic steps often increased the number of transformations. Reported catalytic syntheses of **17** typically required more than 20 steps, indicating that the synthesis of leucascandrolide A remains a formidable challenge.

Our retrosynthetic analysis is outlined in Figure 8. The macrolactone core of leucascandrolide A (**17**), a known intermediate of **24**, would be derived from two fragments of comparable size, **25** and **26**, via disconnection of the C7–C8 bond. A boron enolate‐mediated aldol reaction was envisioned to couple these fragments with high diastereoselectivity through 1,5‐asymmetric induction. Fragment **25** would be prepared from alcohol **27** containing a 1,3‐*syn* diol unit, in which the C11 and C12 stereocenters would be established by a CPA‐catalyzed asymmetric crotylation (Table 2). Fragment **26** would be constructed from enantioenriched propargyl silyl ether **7** through the enantioselective allylation of TMS‐substituted acetylenic aldehyde **4b** with allylic boron reagent **5a** in our CPA/CuBr cocatalyst system.

**SCHEME 7:**
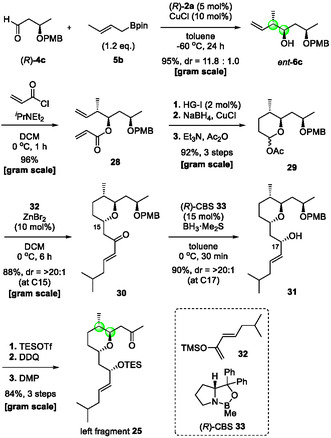
Preparation of left fragment **25**.

The asymmetric crotylation reaction of enantioenriched aldehyde (*R*)‐**4c** under the CPA/CuCl cocatalyst system afforded 1,3‐*syn* diol derivative *ent*‐**6c** in excellent yield with fairly good diastereoselectivity (Scheme 7). Alcohol *ent*‐**6c** was acylated with acryloyl chloride to give ester **28** in 96% yield. Subsequent intramolecular olefin metathesis of **28** using Hoveyda–Grubbs‐I catalyst afforded the corresponding unsaturated lactone quantitatively. Consecutive 1,4‐ and 1,2‐reductions of the lactone under the NaBH_4_/CuCl system, followed by acetylation, gave acetal **29** on a gram scale. The Mukaiyama aldol‐type reaction of **29** with silyl enol ether **32** under mild Lewis acidic conditions proceeded smoothly to furnish enone **30** in high yield with excellent diastereoselectivity, likely through an axial attack on the oxocarbenium intermediate. Corey‐Bakshi‐Shibata (CBS) reduction of **30** delivered 1,3‐*syn* allylic alcohol **31** in 90% yield (dr > 20:1 at C17). Finally, protection of the secondary alcohol with triethylsilyl (TES), oxidative PMB removal with 2,3‐Dichloro‐5,6‐dicyano‐1,4‐benzoquinone (DDQ), and subsequent Dess–Martin oxidation provided ketone **25** in excellent yield. Left fragment **25** could be prepared in only 10 steps from aldehyde (*R*)‐**4c** in 55.9% overall yield (94.4% average yield).

**SCHEME 8:**
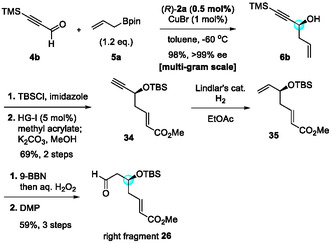
Preparation of right fragment **26**.

Next, we synthesized right fragment **26** by using CPA/CuBr‐catalyzed allylboration (Scheme 8). After further optimization of the reaction conditions, propargyl alcohol **6b** was obtained in nearly quantitative yield with perfect enantioselectivity, even when the catalyst loading was reduced from 2 mol% to 0.5 mol%. Alcohol **6b** was converted into terminal alkyne **34** in 69% yield via a two‐step transformation—cross metathesis with methyl acrylate using the Hoveyda–Grubbs–I catalyst, followed by mild deprotection of the TMS group—in one pot. Selective hydrogenation of **34** with Lindlar's catalyst and subsequent hydroboration–oxidation sequence gave right fragment **26** in 59% yield over three steps.

**SCHEME 9:**
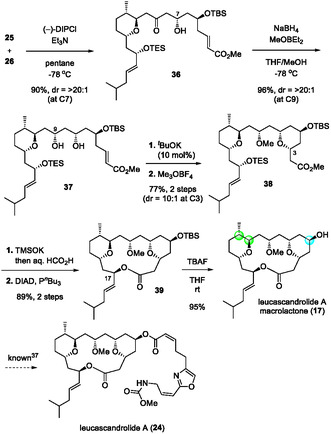
Total synthesis of leucascandrolide A macrolactone (**17**).

With multigram quantities of fragments **25** and **26** in hand, diastereoselective aldol coupling was performed (Scheme 9). Generation of boryl enolate from **25** using (–)‐DIPCl, followed by the addition of **26** at –78°C, furnished aldol adduct **36** in 90% yield with excellent diastereoselectivity. Narasaka–Prasad reduction of the ketone moiety in **24** with diethylmethoxyborane/NaBH_4_ gave 1,3‐*syn* diol **37** in 96% yield. Treatment of **37** with ^*t*^BuOK in THF promoted the intramolecular oxy‐Michael addition to construct 2,6‐*cis*‐tetrahydropyran. The residual alcohol was methylated using Meerwein's reagent to give **38**, which, upon hydrolysis of methyl ester and TES deprotection, provided the corresponding seco acid. Mitsunobu macrolactonization of the crude seco acid efficiently furnished macrolactone **39** with inversion at C17 in 89% yield in two steps. Finally, TBS deprotection by TBAF afforded leucascandrolide A macrolactone (**17**) [91]. This synthetic route furnished macrolactone **17** in 31.2% overall yield over 17 steps and 93.4% average yield from aldehyde (*R*)‐**4c**. The CPA/CuX‐catalyzed allylation and crotylation reactions enabled a scalable and highly stereoselective route, providing the most efficient total synthesis of **17** reported to date.

### 3.3 Scalable Total Synthesis of Bastimolide A

**FIGURE 9:**
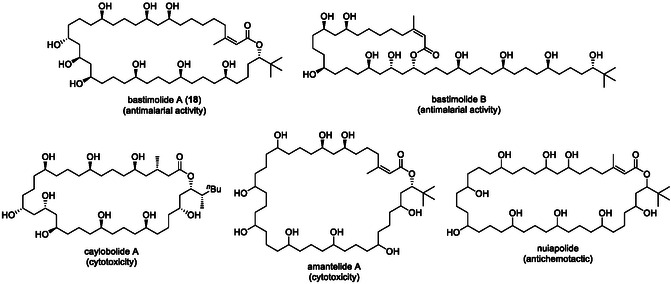
Bastimolide A (**18**) and related 1,5 polyol macrolides.

**SCHEME 10:**
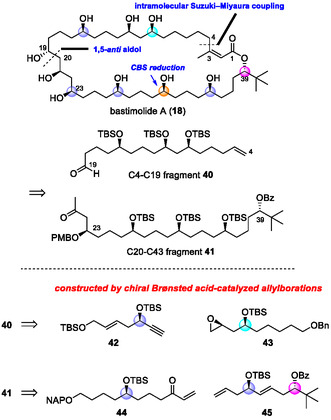
Retrosynthetic analysis of bastimolide A (**18**).

Bastimolide A (**18**) is a 40‐membered macrolide isolated by Gerwick and coworkers. It exhibits potent antimalarial activity (IC_50_ = 80–270 nM), as shown in Figure 9 [108, 109, 110, 111]. Its *α*,*β*‐unsaturated macrolactone, 1,3,5‐triol, and 1,5‐polyol motifs make it a formidable synthetic target. This polyol macrolide has attracted much interest not only for its potent antimalarial activity, but also for SAR studies. Several related natural products with similar structural features have been isolated. However, their complete structures remain undetermined, underscoring the need for synthetic methodologies that can precisely control the stereochemistry of multiple hydroxyl groups (Figure 9) [112]. Although Smith's group achieved an elegant 20‐step total synthesis (0.65% yield) from enantioenriched epoxides, 40 the use of stoichiometric heavy‐metal reagents limited sustainability. To overcome these issues, we designed a catalytic, enantioselective route based on chiral Brønsted acid‐catalyzed C—C bond‐forming reactions with predictable stereocontrol. In our retrosynthetic analysis, we envisioned macrolactone closure via intramolecular Suzuki–Miyaura coupling under mild conditions to preserve the *Z*‐alkene geometry (Scheme 10) [111].

**SCHEME 11:**
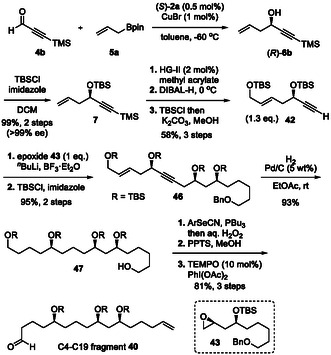
Preparation of C4–C19 fragment **40**.

Although Smith's group achieved an elegant 20‐step total synthesis (0.65% yield) from enantioenriched epoxides [113], the use of stoichiometric heavy‐metal reagents limited sustainability. To overcome these issues, we designed a catalytic, enantioselective route based on chiral Brønsted acid‐catalyzed C—C bond‐forming reactions with predictable stereocontrol. In our retrosynthetic analysis, we envisioned macrolactone closure via intramolecular Suzuki–Miyaura coupling under mild conditions to preserve the *Z*‐alkene geometry (Scheme 9). We expected that the C19 stereocenter would arise from a late‐stage 1,5‐*anti*‐aldol reaction of C4–C19 fragment **40** with C20–C43 fragment **41**. Fragments **40** and **41** would be prepared from simple enantioenriched subunits **42**–**45**, which could be efficiently synthesized by using chiral Brønsted acid‐catalyzed allylation and crotylation. The synthesis commenced with the CPA/CuBr‐catalyzed enantioselective allylboration of TMS‐substituted aldehyde **4b**, affording propargylic alcohol (*R*)‐**6b** in quantitative yield and with perfect enantioselectivity (Scheme 11). Only 0.5 mol% of CPA (*S*)‐**2a** was required in the reaction, and CPA (*S*)‐**2a** was recovered in 98% yield and reused without any loss of catalyst activity. Subsequent TBS protection, olefin metathesis, reduction, and desilylation furnished alkyne **42** in good yield. The nucleophilic ring‐opening reaction of epoxide **43** with alkyne **42**, followed by TBS protection, gave coupling product **46** in excellent yield. Next, selective deprotection of the benzyl (Bn) group was performed under hydrogenation conditions to afford a primary alcohol together with the reduction of alkene and alkyne moieties. Successive Grieco–Nishizawa olefination, selective deprotection of the primary TBS group, and oxidation of the generated alcohol provided aldehyde **40** (C4–C19 fragment) in 41% overall yield in 11 steps.

**SCHEME 12:**
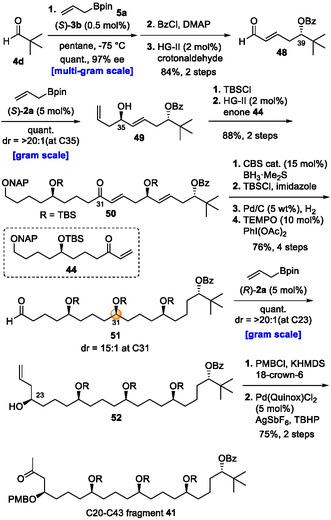
Preparation of C20–C43 fragment **41**.

The synthesis of fragment **41** started with the phosphoramide‐catalyzed allylboration of pivalaldehyde (**4d**) with allylboronate **5a**, affording the corresponding alcohol on a multigram scale (Scheme 12). Notably, the catalyst used in this reaction could be readily recovered in over 95% yield through a simple work‐up procedure. After protection of the generated alcohol with the benzoyl (Bz) group, cross‐metathesis and subsequent asymmetric allylboration of the generated *α*,*β*‐unsaturated aldehyde in the presence of CPA (*S*)‐**2a** furnished homoallylic alcohol **49** with high diastereoselectivity. After TBS protection of the hydroxyl group in compound **49**, olefin metathesis with enone **44** afforded coupling product **50** in very good yield. Subsequent four‐step functional group transformation, including a CBS reduction, furnished aldehyde **51** in 76% overall yield (dr = 15:1). Remarkably, when the asymmetric allylation of this highly functionalized aldehyde was carried out, the desired product was obtained with excellent diastereoselectivity despite the presence of multiple functional groups. Finally, PMB protection of the hydroxyl group, followed by the modified Wacker oxidation, completed the synthesis of C20–C43 fragment **41** in 42% overall yield in 13 steps.

**SCHEME 13:**
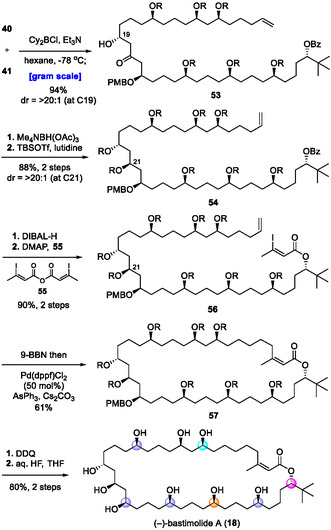
Total synthesis of (–)‐bastimolide A (**18**).

The assembly of fragments **40** and **41** through the Cy_2_BCl/Et_3_N‐mediated diastereoselective aldol reaction gave adduct **53** in 94% yield with excellent diastereoselectivity (Scheme 13). The Evans–Saksena reduction of **53** produced the 1,3‐*anti* diol, which was protected by TBSOTf to give intermediate **54**. Selective removal of the benzoyl group and acylation by using acid anhydride **55** afforded ester **56** having all the necessary carbons of bastimolide A. Intramolecular Suzuki–Miyaura coupling under the optimized conditions furnished macrolactone **57** in 61% yield without *Z*‐olefin isomerization. Finally, global deprotection with DDQ and aq. HF gave bastimolide A (**18**) in 80% yield in two steps. This synthesis provided **18** in 15.4% overall yield in 21 linear steps (91.5% average yield), producing more than 200 mg of **18** from simple starting materials [92]. Our strategy showcases the power of CPA‐ and phosphoramide‐catalyzed enantio‐ and diastereoselective allylborations for the efficient and scalable construction of architecturally complex macrolides.

## 4 Summary and Outlook

We achieved efficient and scalable syntheses of biologically active compounds through asymmetric allylborations mediated by chiral phosphoric acid and chiral phosphoramide catalysts. In pursuing scalable synthesis, we established catalytic methods for constructing synthetically valuable chiral building blocks that were previously difficult to access with conventional approaches, using only a small amount of catalyst. In particular, detailed analysis of TSs associated with chiral phosphoramide catalysis revealed multiple noncovalent interactions characteristic of these catalysts, thereby providing concepts for the principle design of asymmetric catalysis to develop reactions suitable for catalytic and scalable synthesis. Future advances in the design of catalytic asymmetric reactions based on multipoint interactions will pave the way for scalable syntheses of structurally complex and biologically important natural products and pharmaceutical agents, ultimately contributing to drug development.

## Author Contributions

**Shigenobu Umemiya** contributed conceptualization and writing original draft. **Masahiro Terada** contributed writing review and editing, funding acquisition, and supervision.

## Funding

This work was supported by the Grant‐in‐Aid for Scientific Research (C) (23K04730), a Grant‐in‐Aid for Scientific Research on Innovative Areas (JP17H06447), and a Grant‐in‐Aid for Transformative Research Areas (A) (JP23H04908).

## Conflicts of Interest

The authors declare no conflicts of interest.

## Data Availability

Data sharing is not applicable to this article as no new data were created or analyzed in this study.
